# 

**Published:** 1908-11

**Authors:** 


PUBLISHED MONTHLY. SUBSCRIPTION $1.00

ATLANTA

### JOURNAL-RECORD OF MEDICINE

Successor to { Atlanta Medical and Surgical Journal, Established 1855. and Southern Medical Record, Established 1870. } 53d Year

Vol. X. ATLANTA, GA., NOVEMBER, 1908. No. 8

